# End-of-life decision disparities according to the gross national income in critically ill patients: a secondary analysis of the ETHICUS-2 study

**DOI:** 10.1186/s13613-025-01419-1

**Published:** 2025-03-08

**Authors:** Ignacio Martin-Loeches, Charles L. Sprung, Eric Wolsztynski, Rachael Cusack, Suzana Margareth Lobo, Alessandro Protti, Alexander Avidan

**Affiliations:** 1 Department of Intensive Care Medicine, Multidisciplinary Intensive Care Research Organization (MICRO), St James’ Hospital, Dublin, D08 NHY1 Ireland; 2 Department of Anesthesiology, Critical Care and Pain Medicine, Hadassah Medical Organization and Faculty of Medicine, Hebrew University of Jerusalem, Jerusalem, Israel; 3 School of Mathematical Sciences, University College Cork, Western Gateway Building, Western Road, Cork, T12 XF62 Ireland; 4 Insight SFI Centre for Data Analytics, University College Cork, Western Gateway Building, Western Road, Cork, T12 XF62 Ireland; 5 Unidade de Terapia Intensiva, Hospital de Base, Faculdade de Medicina de São José Do Rio Preto, São José do Rio Preto, SP Brasil; 6 IRCCS Humanitas Research Hospital, Rozzano, Milano Italy; 7 Department of Biomedical Sciences, Humanitas University, Pieve Emanuele, Milano Italy

**Keywords:** Intensive care, End-of-life, Hospital mortality, Gross national income, Life-sustaining measures

## Abstract

**Aim:**

This study aimed to evaluate the association of end-of-life decisions and time to death in a global cohort of critically ill patients who participated in the international study on end-of-life practices in intensive care units (ICU) (Ethicus-2 study).

**Methods:**

A post hoc analysis was conducted on data from a worldwide observational study that prospectively recruited adult ICU patients who died between September 1, 2015, and September 30, 2016, from 199 ICUs in 36 countries.

**Results:**

The end-of-life pathways of 10,547 ICU non-survivors were s analysed. Patients in high-income countries exhibited a significantly shorter time to death compared to those from middle-income countries. Additionally, therapeutic decisions were found to have a significant but varied association with the length of ICU stay across gross national income (GNI) groups. Specifically, patients in high-income countries with no decision had the shortest length of stay (LOS) overall. However, withdrawing or withholding life-sustaining treatment led to longer LOS in both middle and high GNI countries.

**Conclusion:**

This study’s findings highlight the need for uniformity in global end-of-life decision-making. Outcomes are significantly associated with gross national income (GNI). Moreover, patients in high-income nations tend to have shorter ICU stays before death.

## Take home message

End-of-life decision-making is very different according to the regions, and in high-income nations, patients stay shorter preceding death.

## Introduction

Global mortality rates in intensive care units (ICUs) average between 10 and 25% [1, 2]. Acute illness often accounts for mortality shortly after ICU admission, whereas patients who succumb after an extended length of stay (LOS) typically develop complications leading to delayed mortality [3–5]. The accumulation of multimorbidity, organ failure, and ICU-related complications can be anticipated [6], prompting physicians to engage in decision-making processes regarding end-of-life care, frequently involving the withholding or withdrawing of life-sustaining measures [6].

The organisation of ICUs plays a crucial role in determining morbidity and mortality [8, 9]. Previous studies have revealed an inverse correlation between in-hospital mortality and a country’s gross national income (GNI) [10]. Moreover, GNI positively correlates with increased ICU or hospital LOS preceding death [6].

Thanks to significant advances in modern medicine, it is now possible to sustain and support vital organ function beyond the point where a patient could be expected to return to an acceptable quality of life. Consequently, managing the dying process has become necessary for physicians and other ICU team members, including nurses and social workers [7]. However, end-of-life decisions in the ICU are complex processes. Decision-makers must consider several factors, including illness severity, physiological reserve, pre-morbid conditions, frailty, patients’ wishes, and family beliefs [1]. The limitation of life-sustaining therapy or refocusing patient care goals toward comfort and dignity in those unlikely to survive their illness is a common practice in the ICU [8–12]. Up to 12% of health expenditure in high-income countries is allocated to less than 1% of people who die in a given year [13]. The results of the Ethicus-2 study identified significant worldwide variations in patterns of end-of-life management [14]. We hypothesised that there is an association between GNI and end-of-life decisions and practices worldwide.

## Materials and methods

This study constitutes a pre-defined post hoc analysis of data collected globally during end-of-life practices in intensive care units (ICUs) as part of the Ethicus-2 study [14]. Ethicus-2 was a prospective, multinational, observational study that included consecutive admissions to adult ICUs worldwide. Patients who died or had any limitation of life-sustaining treatment were included in the study. The limitation of life-sustaining therapy was defined as the withholding or withdrawing of life-sustaining medical interventions or active shortening of the dying process [14]. The study encompassed patients admitted to 199 ICUs across 36 countries on five continents. Full details regarding the methodology and definitions have been previously published [14].

### Patients

Non-survivors with complete data on ICU mortality and time-to-death available were included in the analysis. Patients who remained alive after 60 days in the ICU were considered survivors for this analysis. Patients were categorised into three groups based on time-to-death following ICU admission: early (< 5 days), intermediate (5–28 days), and late (> 28 days). The cut-off for early mortality was determined based on the median mortality time in our population.

### Gross national income regions

Gross national income levels were based on the World Bank Atlas Method using threshold income values. High, upper-middle, and lower-middle income levels were defined as GNIs over 12,476 USD, between 4036 USD and 12,475 USD, and between 1026 USD and 4035 USD per capita. No low-income countries were represented in the study.

### Outcome measures

End-of-life decisions were categorised into five categories [12]

1. Withholding: no escalation of current therapy or implementation of further intervention (WH).
2. Withdrawing: removal of current ongoing life-sustaining therapy (WD).
3. Active shortening of the dying process: undertaking an act to reduce time to death (SDP).
4. Brain death (BD).
5. Failed cardiopulmonary resuscitation (CPR).

A hierarchical categorisation was employed for the most functional limitation if more than one occurred (active shortening of the dying process > withdrawing > withholding).

### Ethics

Each participating centre waived informed consent and obtained institutional ethics committee approval. Countries and centres were coded anonymously, and study participants were numbered consecutively to ensure confidentiality. This enabled clinicians to report practices without risking legal liability. Further details regarding Institutional Review Board (IRB) approval can be found elsewhere [14].

### Statistical analysis

Data were presented as means with standard deviation, medians, interquartile ranges, or numbers and percentages. Differences in distribution means were assessed using a t-test or a nonparametric Mann–Whitney test for continuous variables. At the same time, survival patterns across GNI groups and decision types were inspected using stratified Kaplan–Meier survival curve estimates. Differences between groups regarding variable distribution were evaluated using analysis of variance, chi-square test, or Fisher’s exact test as appropriate. All reported p-values were two-sided, and a value lower than 0.05 was considered statistically significant. Measures of association were presented as odds ratios with their 95% confidence intervals.

Multivariate mixed-effects Cox analyses were conducted to determine the relative risk of decision type and GNI group on the risk of a shorter ICU stay duration. For these analyses, lower-middle and upper-middle classes were combined into a single ‘middle GNI’ group to mitigate imbalance across income groups. Interactions between the “income group” (high vs middle-income level based on GNI) and the “decision type” (“no decision”, “withdraw/shorten”, “withhold”) regarding life-sustaining treatment were included as variables in the model. Cox models were adjusted to account for the country effect as a random effect, and p-values were adjusted for the false discovery rate.

Data were analysed using SPSS Statistics software, version 27 for Windows (IMP Corp., Armonk, NY), and R version 4.0.4.

## Results

The present analysis included 12,850 patients. After excluding patients who survived more than 60 days (n = 2303), data from 10,547 patients were analysed.

### Demographics and acute and chronic diagnosis

The mean age of the non-survivors was 66 (SD ± 16) years, with the majority being male in a 3:2 ratio. The leading acute diagnoses for ICU admission were respiratory (38%), cardiovascular failure (28%), and sepsis (26%). Withholding and withdrawing were most associated with acute respiratory failure on admission (n = 1681, 42% and n = 1514, 38%, respectively). Withholding and withdrawing life-sustaining interventions were the dominant end-of-life pathways demonstrated in the analysis of chronic diseases, ranging from 74% for digestive system disease to 86% for those with cancer. The most common chronic comorbidity was cardiovascular (54.3%). Failed CPR was next most common, followed by brain death and, finally, the shortening of the dying process. Those with no chronic comorbid diseases were more likely to die because of failed CPR (20%) or brain death (19%) than those with one or more chronic illnesses. Withholding of further life-sustaining measures was most common in non-survivors with three or more comorbidities (43% vs 40% withdrawal, 14% shortening of the dying process, p ≤ 0.001).

### Comorbidity and end-of-life

The most common comorbidity reported in patients who died was cardiovascular (54%). Most patients with two or more comorbidities had a decision to withhold or withdraw treatment. Only 10% of patients who died in the ICU had no comorbidities, and 60% of these had a decision to withhold or withdraw treatment.

### Gross national income analysis

Most of the study population (9017, 85.5%) were from high-income countries, 1373 (13%) from upper-middle-income countries, and 157 (1.5%) from lower-middle-income countries. There were no contributing centres from low-income countries. In high-income countries, withholding (n = 3362, 37%) and withdrawing (n = 3840, 43%) life-sustaining measures were the most prominent end-of-life pathways. Renal replacement therapy (25%), vasopressors (19%), and tracheal intubation (16%) were most withheld, while vasopressors (15%), invasive mechanical ventilation (12%), and tracheal intubation (8%) were most widely withdrawn. Withholding of life-sustaining measures (n = 585, 42.6%) and failed CPR (n = 482, 35.1%) were the most common end-of-life pathways in the upper-middle income group. Failed CPR (n = 111, 70.7%) was the most common end-of-life pathway in the lower-middle income group. No patients from the lower-middle income group had a shortening of the dying process. Further data can be found in Tables 1 and 2.

**Table 1:** End-of-life pathways and patient demographics

Variables	Total study cohort	End of life pathway					p-value
		Withhold	Withdraw	Shortening of dying process	Failed CPR	Brain death	
Total	(n = 10,547)	(n = 3972)	(n = 4092)	(n = 58)	(n = 1777)	(n = 648)	
Age	66.03 ± 15.9	67.86 ± 15.4	66.97 ± 14.7	66.66 ± 13.0	63.86 ± 17.1	54.71 ± 17.7	< 0.001
Female	4194 (39.8)	1587 (37.8)	1588 (37.9)	21 (0.5)	710 (16.9)	288 (6.9)	0.096
Male	6353 (60.2)	2385 (37.5)	2504 (39.4)	37 (0.6)	1067 (16.9)	360 (5.7)	0.096
Acute issues on admission							
Neurological	2303 (21.8)	706 (30.7)	966 (41.9)	10 (0.4)	196 (8.5)	425 (18.5)	< 0.001
Respiratory	3968 (37.6)	1681 (42.4)	1514 (38.2)	25 (0.6)	678 (17.1)	70 (1.8)	< 0.001
Cardiovascular	2944 (27.9)	1024 (34.8)	1178 (40.0)	14 (0.5)	592 (20.1)	136 (4.6)	< 0.001
Gastrointestinal	1290 (12.2)	509 (39.5)	562 (43.6)	11 (0.9)	197 (15.3)	11 (0.9)	< 0.001
Metabolic	2225 (21.1)	911 (40.9)	884 (39.7)	20 (0.9)	379 (17.0)	31 (1.4)	< 0.001
Surgical	2436 (23.1)	937 (38.5)	946 (38.8)	20 (0.8)	421 (17.3)	112 (4.6)	0.002
Haematological	482 (4.6)	242 (50.2)	159 (33.0)	3 (0.6)	74 (15.4)	4 (0.8)	< 0.001
Trauma	359 (3.4)	86 (24.0)	136 (37.9)	1 (0.3)	55 (15.3)	81 (22.6)	< 0.001
Sepsis	2776 (26.3)	1204 (43.3)	1004 (36.2)	20 (0.7)	535 (19.3)	13 (0.5)	< 0.001
Other	500 (4.7)	183 (36.6)	199 (39.8)	2 (0.4)	107 (21.4)	9 (1.8)	< 0.001
Comorbidities							
Cardiovascular	5730 (54.3)	2256 (39.4)	2235 (39.0)	32 (0.6)	955 (16.7)	252 (4.4)	< 0.001
Neurological-cognitive-muscular	1663 (15.8)	637 (38.3)	715 (43.0)	8 (0.5)	209 (12.6)	94 (5.7)	< 0.001
Chest disease	2373 (22.5)	909 (38.3)	981 (41.3)	15 (0.6)	372 (15.7)	96 (0.4)	< 0.001
Kidney and urinary systems	1427 (13.5)	604 (42.3)	551 (38.6)	9 (0.6)	241 (16.9)	22 (1.5)	< 0.001
Digestive system	1114 (10.6)	443 (39.8)	492 (44.2)	8 (0.7)	148 (13.3)	23 (2.1)	< 0.001
Immunologic	812 (7.7)	384 (47.3)	265 (32.6)	4 (0.5)	139 (17.1)	20 (2.5)	< 0.001
General history	3651 (34.6)	1513 (41.4)	1421 (38.9)	16 (0.4)	564 (15.4)	137 (3.8)	< 0.001
Cancer	1594 (15.1)	749 (47.0)	621 (39.0)	13 (0.8)	192 (12.0)	19 (1.2)	< 0.001
Unknown	240 (2.3)	71 (29.6)	62 (25.8)	1 (0.4)	72 (30.0)	35 (14.2)	< 0.001
Number of comorbid							
0	1045 (9.9)	264 (25.3)	366 (35.0)	9 (0.9)	208 (19.9)	198 (18.9)	< 0.001
1	3793 (36.0)	1342 (35.4)	1451 (38.3)	16 (0.4)	713 (18.8)	271 (7.1)	< 0.001
2	3181 (30.2)	1270 (39.9)	1272 (40.0)	16 (0.5)	496 (15.6)	127 (4.0)	< 0.001
> 3	2528 (24.0)	1096 (43.4)	1003 (39.7)	17 (0.7)	360 (14.2)	52 (2.1)	< 0.001
Reason for decision							
Sepsis/shock	2914 (27.6)	1588 (54.5)	1291 (44.3)	20 (0.7)	15 (0.5)	0	< 0.001
Multisystem organ failure	4203 (39.9)	2141 (50.9)	2016 (48.0)	34 (0.8)	12 (0.3)	0	< 0.001
Neurologic	3037 (28.8)	1210 (39.8)	1788 (58.9)	30 (1.0)	6 (0.2)	3 (0.1)	< 0.001
Chronic disease	3821 (36.2)	1979 (51.8)	1807 (47.3)	29 (0.8)	6 (0.2)	0	< 0.001
Poor quality of life	3391 (32.2)	1537 (45.3)	1819 (53.6)	28 (0.8)	6 (0.2)	1 (0.03)	< 0.001
Unresponsive to maximal therapy	4898 (46.4)	2495 (50.9)	2350 (48.0)	35 (0.7)	17 (0.3)	1 (0.02)	< 0.001
Age	1007 (9.5)	555 (55.1)	443 (44.0)	7 (0.7)	2 (0.2)	0	< 0.001
Patient request	939 (8.9)	470 (50.1)	463 (49.3)	5 (0.5)	0	1 (0.1)	< 0.001
Family request	1772 (16.8)	817 (46.1)	937 (52.9)	17 (1.0)	0	1 (0.1)	< 0.001

Data are presented as the mean ± standard deviation (SD) or as the number of patients with the percentage in parenthesis, as appropriate

**Table 2:** End-of-life pathways and gross national income

	Total study cohort	Gross national income			p-value
		High income	Upper-middle income	Lower-middle income	
Total	(n = 10,547)	(n = 9017)	(n = 1373)	(n = 157)	
End-of-life pathway					
Withhold	3972 (37.7)	3362 (37.3)	585 (42.6)	25 (15.9)	< 0.001
Withdraw	4092 (38.8)	3840 (42.6)	235 (17.1)	17 (10.8)	< 0.001
Shortening of dying process	58 (0.5)	53 (0.6)	5 (0.4)	0	0.395
Failed CPR	1777 (16.8)	1184 (13.1)	482 (35.1)	111 (70.7)	< 0.001
Brain death	648 (6.1)	578 (6.4)	66 (4.8)	4 (2.5)	0.012
Withheld					
Endotracheal tube	1601 (15.2)	1489 (16.5)	102 (7.4)	10 (6.4)	< 0.001
Mechanical ventilation	1465 (13.9)	1358 (15.1)	98 (7.1)	9 (5.7)	< 0.001
Vasopressors	1857 (17.6)	1687 (18.7)	166 (12.1)	4 (2.5)	< 0.001
TPN	536 (5.1)	481 (5.3)	55 (4.0)	0	< 0.001
Enteral feeding	558 (5.3)	498 (5.5)	60 (4.4)	0	< 0.001
IV fluids	350 (3.3)	281 (3.1)	69 (5.0)	0	< 0.001
Dialysis	2466 (23.4)	2270 (25.2)	174 (12.7)	22 (14.0)	< 0.001
Sedation/analgesia	187 (1.8)	153 (1.7)	32 (2.3)	2 (1.3)	0.150
Withdrawn					
Endotracheal tube	843 (8.0)	773 (8.6)	68 (5.0)	2 (1.3)	< 0.001
Mechanical ventilation	1146 (10.9)	1073 (11.9)	68 (5.0)	5 (3.2)	< 0.001
Vasopressors	1445 (13.7)	1359 (15.1)	82 (6.0)	4 (2.5)	< 0.001
TPN	308 (2.9)	288 (3.2)	20 (1.4)	0	< 0.001
Enteral feeding	698 (6.6)	610 (6.8)	88 (6.4)	0	< 0.001
IV fluids	749 (7.1)	654 (7.2)	96 (7.0)	0	0.001
Dialysis	604 (5.7)	564 (6.3)	38 (2.8)	2 (1.3)	< 0.001
Sedation/analgesia	167 (1.6)	138 (1.5)	29 (2.1)	0	0.052

Data are presented as the mean ± standard deviation (SD) or as the number of patients with the percentage in parenthesis, as appropriate. Rates are that of the End-of-Life pathway per income group

### Time to death

**Fig. 1:**
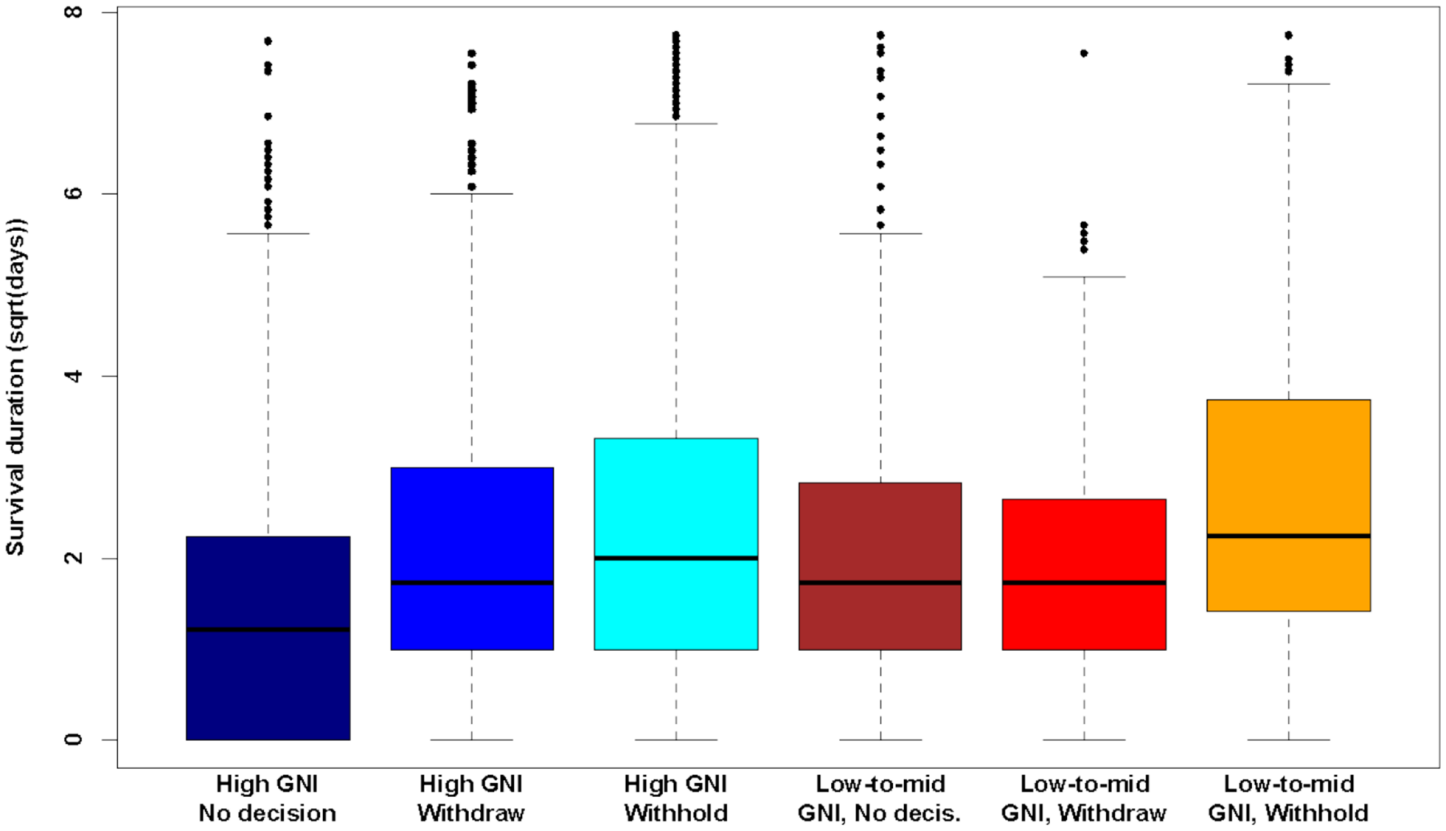
Distribution of ICU stay duration (here in squareroot-time) by income group and decision type

Most patients (n = 5802, 55%) died within the first 5 days in the ICU, 4112 (39%) between days 5–28, and 633 (6%) between days 29–60. A log-rank test showed a statistically significant difference in LOS durations between high-income and middle-income countries (p = 0.0019). Figure 1 analyses the effect of end-of-life decisions on patient mortality, as per the GNI grouping.

### Effect of end-of-life decision on time to death, by GNI

In high-income countries, 6.3% of patients died late (> 28 days) with a withhold or withdraw decision. Up to 50% of the patients died in the first 5 days, and a further 43% had a withhold or withdraw decision within 28 days of admission. In the lower-middle income group, no patients were in ICU longer than 28 days, with 71% dying in the first 5 days with a withhold or withdraw decision and the remaining 29% within 28 days. In the upper-middle income group, 9.2% of patients died after 28 days in ICU with a withhold or withdrawal decision. In this group, 48% of patients who died with a withdraw/withhold decision did so in the first 5 days, with 90% of patients dying within 28 days with a withdraw/withhold decision. Patients in middle GNI countries with a decision to withhold treatment had the most prolonged LOS overall.

For high-income countries, LOS significantly differed between any two types of decisions (p < 0.03 in all cases). Significantly longer LOS was associated with a decision to withdraw (HR = 0.59, p < 0.0001) or withhold (HR = 0.52, p < 0.0001) life-supporting treatment, compared to cases where no decision was made. For middle-income countries, LOS patterns significantly differed between WH and WD decision groups (p = 0.0001). However, no significant difference was observed between the “no decision” and “withdraw support” decision groups (p = 0.4395) in centres in middle-income countries.

### ***Effect of country income level on time to death (***Fig. 2***)***

**Fig. 2:**
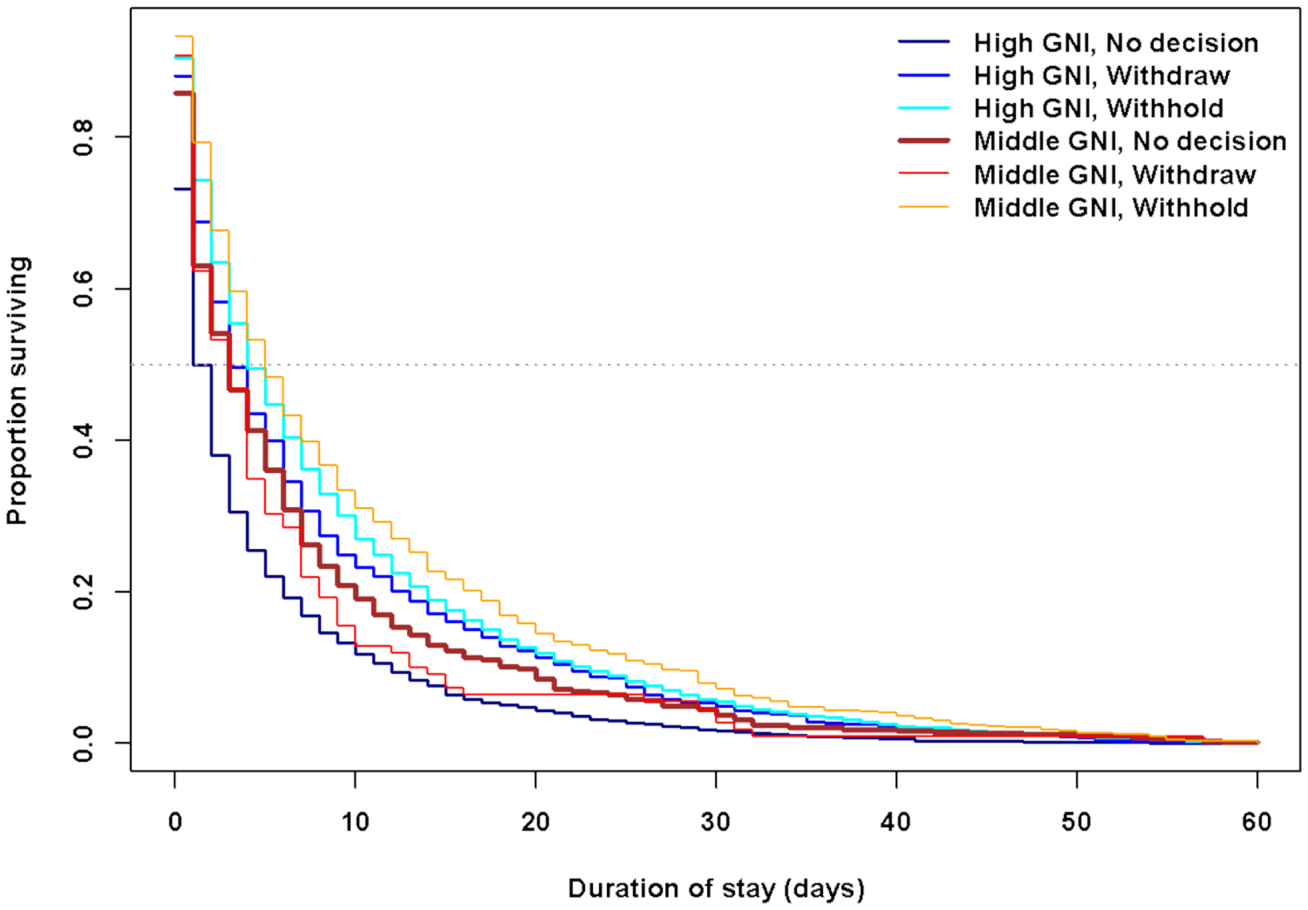
Kaplan–Meier curve illustrating distributions of ICU stay durations per income group and decision type

Results indicated a significant increase in the mortality rate (HR = 1.34, p = 0.0022) in centres from high-income countries compared to middle-income countries for cases where no decision was made. LOS significantly differed between high- and middle-income country centres in cases where a decision to withhold support was made (p = 0.0024), with shorter LOS in high-income countries. No significant difference in LOS was found between high- and middle-income countries in cases where a decision to withdraw support was made (p = 0.0913).

Finally, the analysis indicated significantly reduced mortality rates for middle-income country patients with a decision to withdraw (HR = 0.67, p = 0.0023) or withhold (HR = 0.54, p < 0.0001) life support treatment compared to high-income country patients who withdraw or withhold (Table 3). Likewise, LOS was significantly longer in high-income country centres when a decision was made to withdraw (HR = 0.78, p = 0.0150) or withhold (HR = 0.69, p = 0.0001) life support treatment compared to LOS of patients with no withdraw or withhold decision in middle-income country centres.

**Table 3:** Multivariate mixed-effect Cox proportional hazard model, with country as a fixed effect

Multivariate mixed-effect Cox PH models	HR	Standard Error	p-value
	Baseline profile: high GNI, No decision		
High GNI, withdraw	0.586	0.050	< 0.0001
High GNI, withhold	0.515	0.029	< 0.0001
Middle GNI, no decision	0.748	0.091	0.0016
Middle GNI, withdraw	0.672	0.130	0.0023
Middle GNI, withhold	0.544	0.088	< 0.0001
	Baseline profile: middle GNI, No decision		
High GNI, no decision	1.338	0.091	0.0022
High GNI, withdraw	0.784	0.097	0.0150
High GNI, withhold	0.689	0.088	0.0001
Middle GNI, withdraw	0.899	0.110	0.3400
Middle GNI, withhold	0.728	0.058	< 0.0001

## Discussion

The results of this study underscore the lack of consistency in global end-of-life decision-making, a phenomenon significantly shaped by gross national income (GNI). Furthermore, patients in high-income countries typically experience shorter stays in the ICU before death. Notably, individuals for whom a decision to withhold or withdraw life support was made had longer ICU stays in both high and middle-income countries compared to those without such choices. Interestingly, patients in middle-income countries for whom life support measures were withheld had longer ICU stays compared to other patient profiles in both middle and high-income countries.

The interplay between comorbidity and chronic disease among patients admitted to the ICU may influence the early decision to withhold or withdraw treatment, particularly in higher-income countries [15]. Patients in wealthier nations may endure longer with more significant comorbidity, thereby impacting decisions regarding ICU treatment. Notably, lower-middle and upper-middle-income countries exhibit significantly shorter life expectancies than their higher-income counterparts [16]. Consequently, individuals admitted to ICUs in wealthier nations may present with a higher burden of comorbidities due to prolonged survival with chronic illnesses [17]. Socioeconomic variations contribute to increased disease burden, comorbidity, and severity upon ICU admission [18]. However, it is plausible that resources for diagnosing chronic diseases may be lacking in lower-middle-income countries, leading to a reporting bias where data from higher-income countries may overrepresent chronic illness prevalence upon ICU admission [19]. Additionally, it’s essential to acknowledge that severely ill patients who never reach the ICU in resource-challenged countries may contribute to the observed differences in ICU admission patterns.

The prevalence of withholding or withdrawing life-sustaining treatment in higher-income countries was statistically significantly higher than in lower-middle-income countries, with 80% of patients who died in the higher-income group having a withdraw or withhold decision, compared to 56% in the lower-middle group. Additionally, more withholding and withdrawing decisions in high-income countries have been documented in older patients with and without COVID-19 [20, 21]. The findings highlight significant disparities in ICU outcomes and end-of-life decisions across income groups, reflecting the impact of resource availability and ethical practices. In lower-middle-income settings, the high mortality rate within the first 5 days (71%) emphasises limited capacity for prolonged ICU care. This is consistent with Avidan et al. [14], which identified high CPR failure rates in Africa due to systemic resource constraints.

Conversely, middle-income countries showed the most prolonged ICU stays among patients with withheld treatment decisions, potentially indicating delayed transitions to palliative care, as seen in the Ethicus-2 Study [14]. In upper-middle-income countries, 90% of deaths occurred within 28 days of ICU admission after a withdrawal/withhold decision, suggesting better resource availability but raising questions about variability in ethical practices and cultural attitudes toward limiting care [22]. These findings underscore the need for tailored strategies to address inequities in end-of-life care globally. This discrepancy suggests that more patients in higher-income countries are admitted to the ICU towards the end of life, a trend that has been increasing over the past two decades. It is plausible that in wealthier countries, individuals with terminal comorbid diseases survive longer until ICU admission, as has been previously reported in the UK [15]. At this point, they may be evaluated and deemed unsuitable for life-sustaining measures or admitted for a “trial of ICU.”

Additionally, nearly all instances (99%) of withdrawal or withholding of treatment in cases of sepsis and/or multi-organ system failure align with findings by Daviaud et al. who identified sepsis as independently associated with death within 3 days of ICU admission [22]. Other reported findings indicate that ICUs in higher GNI countries tend to have greater bed capacity and admit patients with higher severity of illness [23]. The increased availability of beds in these settings may lead to accepting patients who are more critically ill and less likely to survive [24].

Unfortunately, our dataset lacks information on patients who may have transitioned between the withhold and withdraw groups. The distinction between the two can blur in some instances as physicians deliberate the optimal course of action [25]. Additionally, we have not categorised patients into medical or surgical groups. Significant regional and cultural disparities exist in withholding and withdrawing life-sustaining measures, which could affect how these decisions are reported and implemented [26]. Prior research has indicated that patients who underwent surgery were more likely to have an extended stay in the ICU before succumbing to their illness [27]. Mortality within 30 days of surgery is a global quality indicator and may influence end-of-life decision-making [29].

Drawing definitive conclusions from our findings presents challenges as wealthier healthcare systems, often affiliated with education and research institutions, are overrepresented in our study. Our findings are primarily based on limited data from lower-income regions, underscoring the need for further investigation. Unfortunately, the limited contribution of information from lower-income areas of high-impact medical journals has not changed in the last 20 years [30]. Specifically, whether the disparities observed in end-of-life decisions stem from differences in resources or practices cannot be concluded from the data of this study. Some of the pertinent questions raised by previous analyses of these data still need more detailed information on why certain patients received a decision to withhold or withdraw treatment while others did not. In higher-income countries, patients and families often anticipate death to occur in a hospital setting, whereas this may not hold in lower-income countries [28].

In the United States, for example, 20% of deaths occur in the ICU, with fewer than 15% of patients who could benefit from palliative care passing away at home and 50% expiring in acute hospital settings [29]. Patients with cancer are more inclined to die in acute hospital settings in certain countries like England, Belgium, or Canada, as opposed to the United States [30]. However, within the United States, those patients are more likely to be admitted to an ICU within the last 180 days of life [31]. Studies examining ICU admissions within the last 6 months of life have identified substantial variation in patient characteristics, even among different regions within the United States [32]. Some areas tend to admit patients with limited life expectancy to the ICU, which could influence the results observed in our study [26]. Nevertheless, the escalated intensity of medical intervention in the final 6 months of life does not necessarily correlate with improved survival rates [33].

Previous systematic reviews have underscored the critical role of nursing staff levels in the ICU and their profound impact on patient outcomes [34, 35]. Similarly, the availability of round-the-clock critical care staff has significantly affected patient care within ICU settings [36]. However, studies have indicated that these essential resources may not be as readily accessible in low-middle and upper-middle-income countries [37]. Consequently, the scarcity of these resources in lower GNI countries may influence the patterns of end-of-life decision-making observed in our results.

Furthermore, research has demonstrated that admission to tertiary research or university-affiliated hospitals is linked to improved outcomes for conditions such as stroke and myocardial infarction [38]. This context is crucial for interpreting our findings, as we received a more substantial response from ICUs in higher-income countries. The organisation of ICUs, whether they operate in an open or closed format, their availability of round-the-clock ICU specialists, and nursing ratios all play a significant role in determining ICU outcomes and mortality rates [39, 40]. ICU organisation is contingent upon the availability of resources, including human and technological resources, as well as the hospital’s specific setting [34]. Factors such as the severity of the patient’s illness, recent corticosteroid administration, and comorbidities also impact ICU outcomes [41]. Moreover, previous studies have highlighted the association between death in the ICU and conditions such as sepsis and infection [42]. Therefore, analysing these factors in the context of gross national income (GNI) is imperative to better understand their influence on end-of-life decision-making processes.

### Strengths and limitations

This study had two major strengths. First, it was a large-scale, international prospective study that included 199 intensive care units across 36 countries on five continents. Second, the treating physicians collected the data, and the coordinating centre conducted regular reviews for quality assurance, ensuring high data quality.

However, the study also had several limitations. First, most patients were from high-income countries, with only a few from low-middle-income countries. Despite various efforts, we did not recruit any ICUs from low-income countries. Therefore, the results comparing patients from different income countries may be skewed. On the other hand, to the best of our knowledge, studies that include and compare data from lower- or middle-income countries with those from high-income countries are rare. Second, there was a delay between the end of the study (2016) and the finalisation of this aspect of the study. The study’s large size can explain this, as there was a lack of funding to efficiently proceed with the statistical analysis and complex collaboration with many researchers worldwide to analyse and publish the various aspects of the study. Additionally, the COVID pandemic further contributed to the delay. Although practices may have changed post-COVID or for other reasons, the information remains important and relevant despite the delay.

## Conclusions

The variability in end-of-life decisions across countries underscores the influence of the country’s income level on these decisions. While patients in high-income countries typically experience shorter stays in the ICU before death, further investigation is imperative to understand these disparities and uncover their underlying causes. Optimising the utilisation of ICU services is essential to ensure optimal care for patients in the terminal stages of their disease.

## Data Availability

The data analysed in this study are available upon reasonable request from the corresponding author. The dataset is not publicly accessible due to confidentiality agreements.
